# From applause to disappointment – appreciation among healthcare providers that provided end-of-life care during the COVID-19 pandemic and its impact on well-being – a longitudinal mixed methods study (the CO-LIVE study)

**DOI:** 10.1186/s12913-024-11999-6

**Published:** 2024-12-18

**Authors:** Masha S. Zee, Bregje D. Onwuteaka Philipsen, Erica Witkamp, Yvonne N. Becqué, Anne Goossensen, H. Roeline Pasman

**Affiliations:** 1 Department of Public and Occupational Health, Expertise Center for Palliative Care, Amsterdam UMC, VU University, Van Der Boechorststraat 7, Amsterdam, 1081 BT The Netherlands; 2 Department of Public Health, Erasmus MC University Medical Center, Rotterdam, The Netherlands; 3 Research Center Innovations in Care, Rotterdam University of Applied Sciences, Rotterdam, The Netherlands; 4 University of Humanistic Studies, Utrecht, The Netherlands

**Keywords:** COVID-19, Apprecation, Healthcare providers, End-of-life care

## Abstract

**Background:**

The healthcare sector is facing increasing work pressure, making a healthy workforce essential. Appreciation is a factor influencing well-being, and the COVID-19 pandemic offers valuable insights into this. This study aims to: 1) describe to what extent end-of-life care providers felt appreciated and understood during the first 18 months of the pandemic, 2) examine the impact of appreciation on their well-being, and 3) explore their perceptions of what appreciation should look like.

**Methods:**

A longitudinal mixed methods study among healthcare providers in the Netherlands delivering end-of-life care during the COVID-19 pandemic. Surveys were conducted at four timepoints (*n* = 302), and interviews were conducted at three timepoints (*n* = 17) during the first 18 months of the pandemic. Generalized Estimating Equations analysis was performed on the quantitative data and thematic analysis was conducted on the interview data.

**Results:**

This study shows that feeling of appreciation among healthcare providers peaked in the first wave of the pandemic, but significantly dropped in the second wave, with only about half of the healthcare providers feeling appreciated. This slightly improved afterwards. Furthermore, nearly half of healthcare providers felt misunderstood during the first 18 months of the pandemic. Additionally, this study shows that between September 2020 and September 2021 about 1 in 3 healthcare providers had a score on the Well-Being Index indicating higher risk for burnout. Feeling appreciated and not feeling understood were both significantly associated with worse well-being. Interviews revealed that nurses did not always feel understood and appreciated by society, employers, patients and their families, as well as their own friends and family, leading to feelings of sadness, anger, and frustration. Three major themes emerged: ‘recognizing real needs, ‘we are not in this together’ and ‘short-lived appreciation that failed to lead to structural changes’.

**Conclusions:**

This study shows that during the COVID-19 pandemic, healthcare providers often felt neither appreciated nor understood, which is associated with lower scores of well-being. The expressed appreciation often did meet their needs or expectations, from both employers and society. With healthcare provider well-being still under strain, sustained attention to appreciation and understanding is important for retaining the workforce.

**Supplementary Information:**

The online version contains supplementary material available at 10.1186/s12913-024-11999-6.

## Introduction

Currently, the healthcare sector in the Netherlands, as well as in other European countries, is dealing with continuously rising work pressure due to growing demand and a shortage of healthcare providers [1, 2]. This growing pressure threatens both the quality and accessibility of care [2]. This high work pressure also applies to palliative care, where demand is expected to continue increasing in the near future [3, 4] and the shortage of healthcare professionals is considered one of the biggest challenges in palliative care [4]. This highlights the importance of retaining our current workforce in this field, yet the well-being of healthcare providers is currently under strain.

The COVID-19 pandemic acted as a catalyst for pre-existing problems and, in addition to exposing the resilience and vulnerabilities of the workforce [1], it also revealed new challenges related to the well-being of healthcare providers [5]. There were changes in end-of-life care, due to e.g. visit restrictions and wearing personal protective equipment, requiring healthcare professionals in this field to provide care that differed from the usual practices [6–10], which jeopardized their well-being. Research shows that these professionals experienced increased symptoms of stress, depression, anxiety, post-traumatic stress, and emotional exhaustion [11–16].

Appreciation appears to be an important aspect for the well-being of healthcare providers and can be experienced on different levels, such as appreciation from loved ones, patients, employers and society. Research conducted in the Netherlands shows that 19% of palliative care providers report feeling out of balance due to a lack of appreciation [17]. A study among cancer care physicians indicates that feeling unappreciated or used is associated with burnout [18]. The COVID-19 pandemic is a compelling case to study appreciation of healthcare providers because, during the first wave, healthcare providers experienced a significant surge in appreciation. People brought gifts and food, applauded, bonuses were given, and employers showed their gratitude. This support was mostly due to people being grateful for the selflessness of healthcare providers who saved lives and worked tirelessly [5, 19]. The COVID-19 pandemic seemed like an opportunity to increase the appreciation and recognition of healthcare work [20]. Societal appreciation during the pandemic contributed to the well-being of healthcare providers. In a study on lived experiences, healthcare providers noted that the increased attention during the pandemic allowed their efforts to be better understood and, as a result, more appreciated [21]. A Spanish study demonstrated that when healthcare providers felt positively appreciated by society, this served as a protective factor for the (social) well-being of frontline healthcare workers during both the first and second waves [22]. Additionally, a study of night-shift healthcare workers found that feeling appreciated by the general population during the pandemic had a positive impact on their work-related quality of life [23]. These studies did not further specify what healthcare providers exactly understand as appreciation from society [22, 23].

However, this wave of public appreciation seemed to decline after the first wave [22]. Despite healthcare providers continuing to perform the same tasks and facing a higher workload and changes in their care delivery, the gifts and applause ceased, sometimes shifting to misunderstanding, stigmatization (resulting from the belief that healthcare providers were carriers of the virus), and even aggression towards them [5, 22, 24]. The insufficient appreciation negatively affected the well-being of healthcare providers. In a study on lived experiences, healthcare providers described, on the one hand, that they often felt the public did not fully understand the challenges and significance of their work, which left them feeling frustrated [21]. Another study shows that negative public responses like criticism and discrimination harm healthcare workers' engagement and well-being, while positive responses like encouragement and donations enhance them [25]. Furthermore, healthcare providers experienced frustration that they did not receive the appreciation from organizational structures within the healthcare system after risking their health to care for patients [26, 27]. The appreciation from employers was important for different healthcare workers, such as nurses and doctors during this crisis, but it did not always seem sufficient [23, 27–31]. For instance, in a review on experiences of frontline healthcare providers it was described how healthcare providers wanted recognition for their sacrifices and work. However, it was not always clear what that appreciation should consist of [29].

Since most literature focuses on the early stages of the pandemic, it is important to examine what happened with the appreciation and well-being of healthcare providers who provided end-of-life to persons dying later in the pandemic. Additionally, it would be valuable to explore how end-of-life care providers perceive appreciation and what they believe it should look like as this is hardly reported in the literature. This longitudinal mixed methods study aims to describe: 1) the extent to which healthcare providers who provided end-of-life care to persons dying during the COVID-19 pandemic felt appreciated and understood during the first 18 months of the pandemic, 2) the impact of appreciation and feeling understood on their well-being during this period, and 3) how end-of-life care providers experience appreciation and how feeling understood is part of that experience. This information may be important for better supporting healthcare providers who provide end-of-life care to persons dying in times of a healthcare crisis, such as the impending shortage crisis.

## Methods

This study is part of the CO-LIVE study: a mixed methods study into the experiences of bereaved relatives and healthcare providers that provided end-of-life care during the COVID-19 pandemic [7]. In this study, surveys and interviews were utilized at multiple time points during the pandemic among healthcare providers providing end-of-life care. Findings from interviews in earlier phases were used to refine and inspire the design fgof surveys in subsequent phases. Similarly, survey results from earlier phases helped shape the topics and questions for interviews in later rounds. This iterative process of mutual influence between qualitative and quantitative data collection gives the study its mixed methods character.

### Population & data collection

#### Surveys

Data was collected from a convenience sample of healthcare providers that cared for persons dying during the COVID-19 pandemic (for both COVID and non-COVID patients) during the initial 18 months of the COVID pandemic (March 2020 – September 2021). These healthcare providers came from various professions and from different settings in the Netherlands.

**Fig. 1:**
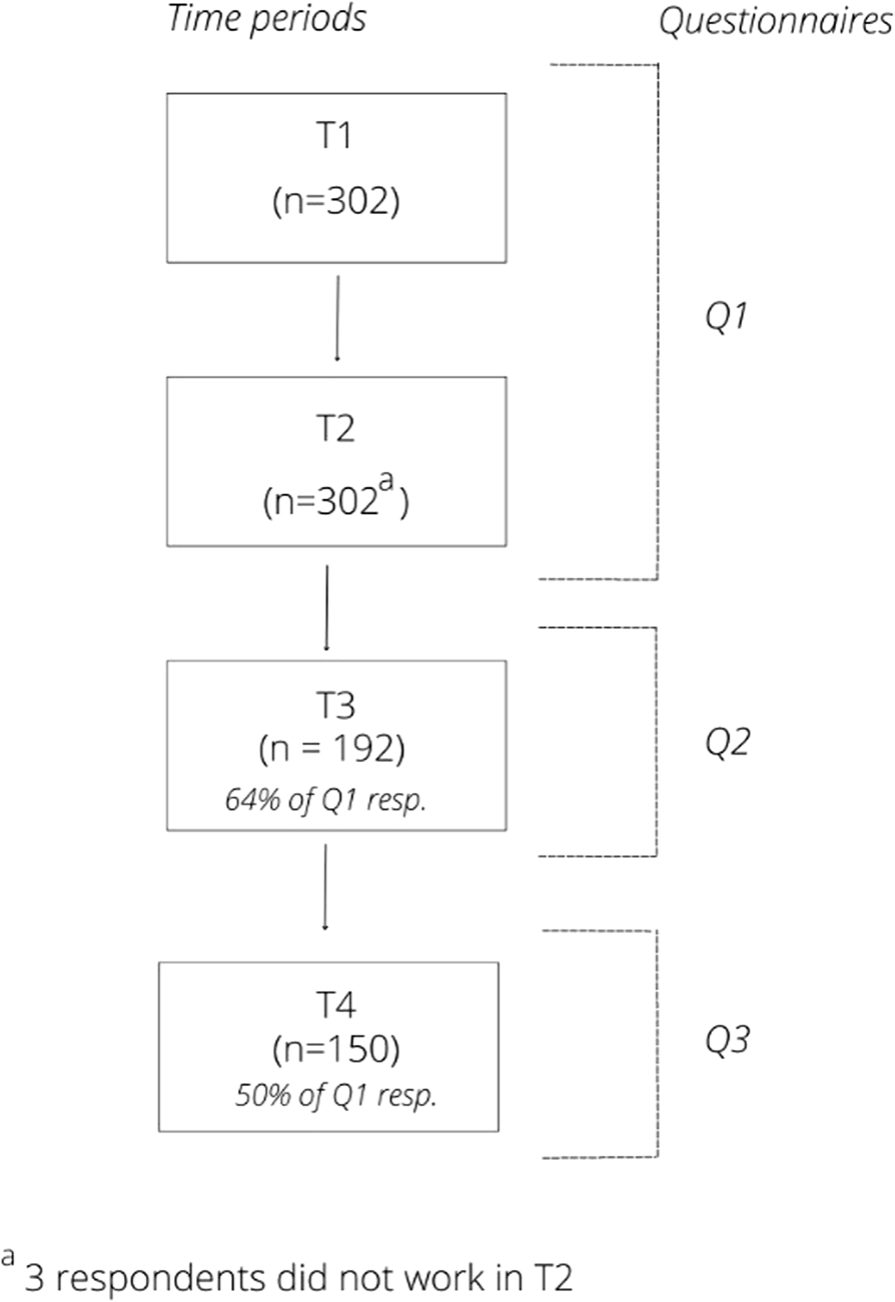
Respondents per time period and questionnaire

**Fig. 2:**
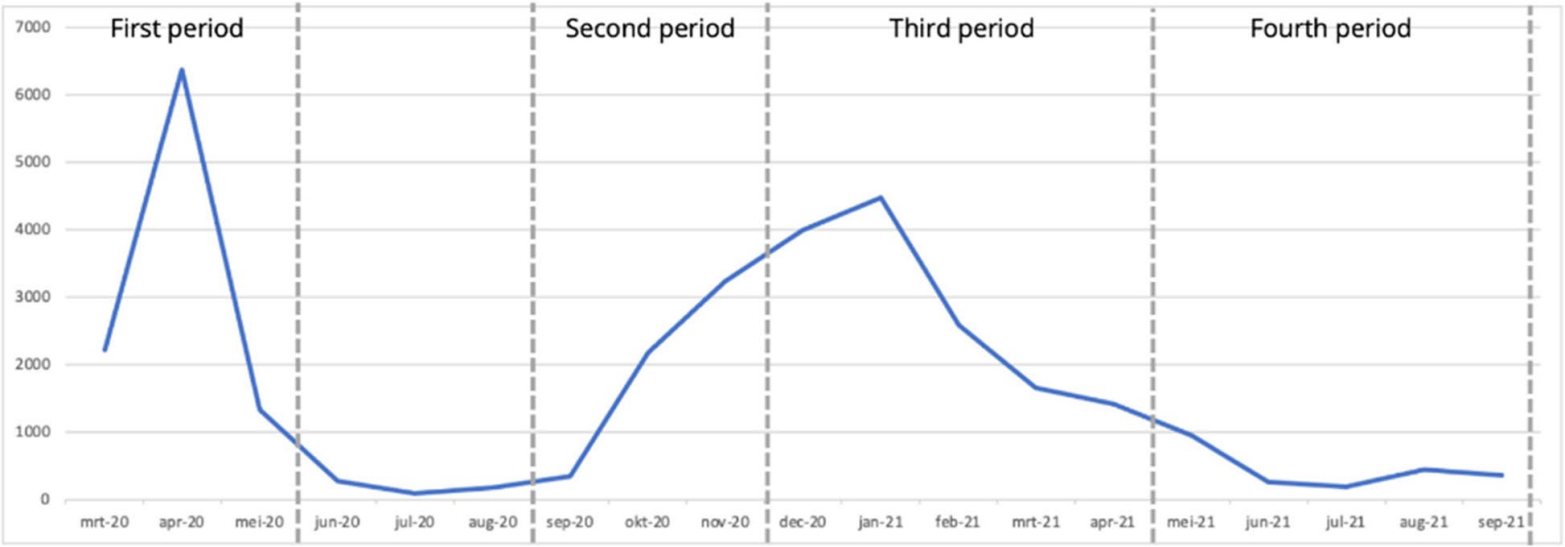
COVID-19 deaths in the Netherlands, march 2020 – September 2021

Data collection covered four time periods, with three questionnaires, Q1, Q2 and Q3 (Fig. 1). Q1 was distributed in November 2020 and contained questions about two time periods: March 2020 – May 2020 (T1) and September 2020 to November 2020 (T2). These periods are considered to be the first wave (T1) and the start of the second wave (T2) of the COVID-19 pandemic in the Netherlands [32, 33]. Q2 was distributed in April 2021 and concerned the period between December 2020 – April 2021 (T3). Q3 was distributed in September 2021 and concerned the time period between May 2021 and September 2021 (T4). Figure 2 shows the number of people that died of COVID-19 in the Netherlands within the four time periods. This provides context about the severity of the pandemic in these researched time periods [34].

Invitations for questionnaire 1 (Q1) were sent to end-of-life care providers that had participated in a previous part of the CO-LIVE study [7]. Additional respondents for Q1 were recruited via (social) media. When respondents indicated in the Q1 questionnaire that we could approach them for another questionnaire, they received an invitation to Q2 and Q3. No other respondents were recruited for Q2 and Q3. Furthermore, respondents of Q1 who missed Q2 could participate in Q3. All questionnaires were designed and distributed via questionnaire software Survalyzer.

#### Interviews

In the survey, respondents were asked whether they would like to participate in the interview study and interview respondents were recruited using maximum variation sampling to ensure diversity in setting and well-being (specifically their Well-Being Index score in T2) via e-mail. An in-depth qualitative longitudinal interview study was conducted with nurses (including nurse aides) of patients who died during the COVID-19 pandemic. The decision was made to conduct the interview study among nurses because they represented the most vulnerable group. The respondents all had experience with end-of-life care during the pandemic. Some worked in settings specifically focused on end-of-life care, such as hospices, while others worked in institutions where end-of-life care was one aspect of their responsibilities, alongside care for other patients, such as in hospitals. While many media reported that healthcare providers experienced mental health issues in practice [35, 36], we did not see this represented in our sample. Therefore, we recruited three additional participants who did not participate in the survey, via social media, networks and previous study participants. This resulted in three extra participants. Two nurse aids were also recruited from an earlier part of the CO-LIVE study to address underrepresentation of this profession [37]. Three rounds of interviews were held with the participants. The first round of interviews took place from March to May 2021, the second from August to September 2021, and the third from January to April 2022. In total, 43 interviews were conducted with 17 participants.

### Measurements & analysis

#### Surveys

Characteristics of respondents included gender, age, profession and setting. Setting was categorized into: home, nursing home, hospice facility, other (including for example a GP practice or institutions for people with intellectual disabilities) and multiple settings. Profession of healthcare providers was divided in three categories; nurses (including registered nurses, nursing aides and nurse practitioners), physicians (e.g. general practitioners, pulmonary and geriatric physicians) and other (e.g. spiritual counselors, paramedics and volunteers).

In the surveys, respondents answered questions about their well-being (T1 – T4) during the COVID-19 pandemic. In T1-T4, respondents were asked to what extent they agreed with the statements: "I feel appreciated as a healthcare provider" and “I have the feeling that people that do not work in healthcare did not understand me” (using a 5-point Likert scale, dichotomized into 'agree' and 'neutral and disagree’. In T2-T4, respondents also completed the Well-being Index [38], a validated questionnaire with 9 questions about the well-being of healthcare providers resulting in a score ranging from -2 (lowest) to + 9 (highest) (Appendix 1). A score of 2 or higher is considered a cutoff score and is associated with a greater risk of burnout, severe fatigue, poor overall quality of life, and moderate or greater intent to leave their current position within the next 24 months. Therefore, the respondents' scores were dichotomized into two groups: those with a score below 2 and those with a score of 2 or higher.

IBM SPSS statistics 28 and Stata 17 were used to analyze the data. Characteristics from healthcare providers and their well-being were described to summarize the data per time period. Generalized Estimating Equations analysis (GEE) was used to study the association between not feeling appreciated and having a WBI score ≥ 2 (OR's). The GEE accounted for clustering of within-subject data (up to four measurements over time per individual). Confounding and effect modification by age, gender, profession, and setting were checked.

#### Interviews

MZ, a female medical anthropologist trained in qualitative research, conducted all interviews via (video) calls due to COVID-19 measures. A topic list, including questions from the survey responses and new experiences, was used (Appendix 2). The interviews, conducted in Dutch, lasted between 21 and 67 min and were audio-recorded.

After conducting the interviews, data were transcribed verbatim and analysed using qualitative data analysis software MAXQDA. We followed the principles of thematic analysis based on a phenomenological approach; focusing on the lived experiences from the respondents [39, 40]. MZ and RP independently coded 16 transcribed interviews from five participants to compare codes and minimize bias. MZ then coded the remaining interviews and discussed the codes with RP, BOP, and EW. MZ grouped the codes into overarching themes, with continuous comparison and discussion among MZ, RP, BOP, and EW. Finally, appropriate quotes were selected by MZ.

### Ethical considerations

Study information was provided with each questionnaire, and respondents gave consent before participation. Interview participants gave verbal informed consent for participation and recording. After transcription, audio recordings were deleted, and all data were anonymized to ensure confidentiality. Personal information and transcripts were stored separately, accessible only to researchers. The Medical Ethics Committee Erasmus MC of Rotterdam assessed that the study did not fall under the Medical Research Involving Human Subjects Act (MEC-2020–0254).

## Results

### Characteristics of healthcare providers

Data of 302 (T1), 299 (T2), 192 (T3) and 150 (T4) respondents is included. The characteristics of the respondents in the different time periods are described in Table 1. Most respondents were women (87.2 – 90.1%) and between 46–60 years of age (44.9—55.8%). Over half of the respondents had a nursing background (61.6 – 71.8%) and about one in third worked in a hospital (27.4 – 30.3%).

**Table 1:** Characteristics of healthcare providers and end-of-life care during four different time periods (absolute numbers and percentage)

	T1	T2	T3	T4
	**Q1**^**a**^		**Q2**	**Q3**
	*N* = 302		*N* = 192	*N* = 150
	N (%)		N (%)	N (%)
**Gender**				
Women	265(89.2)		173 (90.1)	129 (87.2)
Men	32 (10.8)		19 (9.9)	19 (12.8)
**Age**				
⩽35 years	61 (20.7)		30 (15.9)	13 (8.8)
36–45 years	58 (19.7)		29 (15.3)	23 (15.6)
46–60 years	132 (44.9)		96 (50.8)	82 (55.8)
> 60 years	43 (14.6)		34 (18.0)	29 (19.7)
**Profession**				
Nurse	216 (71.8)		129 (68.8)	90 (61.6)
Physician	40 (13.2)		24 (12.8)	22 (15.1)
Other	45 (15.0)		35 (18.6)	34 (23.4)
**Setting**				
Home	47 (15.8)		33 (17.6)	20 (13.7)
Nursing home	64 (21.5)		34 (18.1)	29 (19.9)
Hospital	90 (30.3)		53 (28.2)	40 (27.4)
Hospice facility	54 (18.2)		38 (20.2)	29 (19.9)
Other	17 (5.7)		13 (6.9)	15 (10.3)
Multiple	25 (8.4)		17 (9.0)	13 (8.9)

Number of missing observations range (over Q1-Q3): gender (0–5), age (3–8), profession (0–4), setting (3–5)

^a^Q1 was distributed in T2 and contained questions about both T1 and T2

The characteristics of the interview respondents are included in Appendix 3. All interview respondents were women, with 13 being nurses and 4 being nurse aides. Nurses and nurse aids were represented across various settings, including hospitals, ICU, nursing homes, home care, and hospice facilities, and most participated in 3 interviews.

### Feeling appreciated and understood and well-being of healthcare providers

Table 2 shows how healthcare providers felt appreciated and understood and it shows their well-being in T1-T4. It reveals that healthcare providers felt most appreciated in T1 with 74.3% (difference from T1 and T4 is *p* < 0.05) and significantly least appreciated in T2 with 57.5% (difference from other time periods is *p* < 0.05). In T3, the percentage rises again to 73.8% and then decreases to 67.1% in T4. In all time periods, healthcare providers felt least often appreciated when they worked in hospitals (ranging from 47.7% to 75.6%) and nursing homes (ranging from 51.0% to 61.9%). Additionally, the percentage of healthcare providers feeling not understood during the pandemic remains relatively constant, ranging from 42.1% to 48.1%. There are no significant differences in these percentages. In all time periods, the amount of healthcare providers that felt not understood was significantly lowest in hospice compared to other work settings (ranging from 24.1% to 30.2%).

**Table 2:** Well-being, feeling understood and feeling appreciated during four time periods (absolute numbers and percentage)

	**T1** N (%)	**T2** N (%)	**T3** N (%)	**T4** N (%)
**Felt appreciated**	**220 (74.3)**	**172 (57.5)**	**138 (73.8)**	**98 (67.1)**
Home care	37 (78.7)	33 (73.3)	27 (84.4)	15 (75.0)
Nursing home	39 (61.9)	25 (51.0)	20 (58.8)	16 (55.2)
Hospital	68 (75.6)	42 (47.7)	33 (62.3)	22 (55.0)
Hospice	49 (90.7)	34 (66.7)	33 (86.8)	24 (82.8)
Other	12 (70.6)	23 (62.2)	11 (84.6)	9 (60.0)
More than one	15 (60.0)	13 (50.0)	14 (82.4)	12 (92.3)
**Did not feel understood**	**137 (45.5)**	**135 (45.0)**	**90 (48.1)**	**61 (42.1)**
Home care	19 (40.4)	20 (43.5)	14 (42.4)	9 (47.4)
Nursing home	39 (60.9)	28 (57.1)	22 (64.7)	13 (44.8)
Hospital	43 (47.8)	45 (51.1)	32 (61.5)	21 (52.5)
Hospice	16 (30.2)	14 (27.5)	10 (26.3)	7 (24.1)
Other	6 (35.3)	18 (48.6)	3 (23.1)	7 (46.7)
More than one	12 (48.0)	9 (34.6)	9 (52.9)	4 (30.8)
**WBI ≥ 2**		**101 (35.7)**	**53 (28.8)**	**47 (33.8)**
Home care		16 (35.6)	10 (31.3)	4 (21.1)
Nursing home		23 (50.0)	13 (38.2)	10 (38.5)
Hospital		34 (42.0)	18 (36.7)	17 (47.2)
Hospice		8 (16.3)	3 (8.1)	7 (24.1)
Other		12 (33.3)	5 (38.5)	6 (40.0)
More than one		8 (30.8)	4 (23.5)	3 (23.1)

Furthermore, Table 2 shows that the percentage of healthcare providers with a WBI-score ≥ 2 (indicating a higher risk of burnout, fatigue, and of leaving the profession) is 35.7% in T2, 28.8% in T3, and 33.8% in T4. There were no significant differences between the time periods. In all time periods, healthcare providers most often had a WBI-score ≥ 2 in nursing homes (ranging from 38.2% to 50.0%) and hospitals (ranging from 36.7% to 47.2%), which is a significant difference with healthcare providers in hospice facilities.

#### Association between feeling appreciated and well-being

Table 3 shows the association between feeling appreciated or not feeling understood and well-being during T2-T4. Both adjusted models have been corrected for gender and age. It was also checked for confounding and effect modification by setting and profession, but no confounding or effect modification was found. The OR of having an WBI ≥ 2 was 0.43 for healthcare providers who felt appreciated, indicating an association between well-being and appreciation (Table 3). Additionally, it shows that healthcare providers who did not feel understood by people outside the healthcare sector had 2.15 times higher odds of having a WBI-score of ≥ 2 compared to those who felt understood.

**Table 3:** GEE analysis WBI and feeling appreciated and feeling not understood (ORs and 95% confidence intervals)^a^

	**OR**	**95% BI**	***P*-value**
**Felt appreciated**			
Crude model	0.43	0.30 – 0.62	< 0.001
Adjusted model^b^	0.44	0.30 – 0.65	< 0.001
**Did not feel understood**			
Crude model	2.15	1.47 – 3.13	< 0.001
Adjusted model^b^	2.15	1.47 – 3.15	< 0.001

^a^*N* = up to four observations from 302 respondents; based on Generalized Estimated Equation

^b^The adjusted model has been corrected for gender and age

#### Interviews

In the interviews, we asked nurses if they felt appreciated and understood, and what reasons there were for feeling this way or not. The degree to which they felt appreciated and understood varied among nurses, as did the reasons for not feeling appreciated or understood. The concept of appreciation was explored by asking nurses to describe forms of appreciation from various sources. When they discussed appreciation, it was about appreciation from their loved ones, but especially about their employers (and how the employer could better show appreciation), the patients and their relatives and appreciation they experienced from society. When nurses did not feel appreciated or understood, it made them feel sad, angry, or frustrated. When discussing this topic, three important themes emerged: ‘recognizing real needs, ‘we are not in this together’ and ‘short-lived appreciation that failed to lead to structural changes’ (Appendix 4).

#### Recognizing real needs

##### Appreciation in the first wave

During the first wave, appreciation from the public was mainly shown through applause and kind words on (social) media. During this time, most nurses felt more recognition for their work and the (mental) strain of their profession.

"Society, right… during the first wave we were inundated with all kinds of things: socks, food, fruit, flowers — you name it and we got given it." (R1:nurse, hospital)

However, while they appreciated the kind words, gifts and applause, they did not always find it necessary and sometimes felt uncomfortable because they were simply doing what they always did. This discomfort reflected a tension between external appreciation and their internal sense of simply fulfilling their professional duties.

"Yes, that’s right. Well, the healthcare professionals were the heroes in the first wave, of course, with all that applause, and I was kind of like, ‘come on guys, just be normal. We always just do our job.’ But well, on the other hand it was also a nice feeling." (R12:nurse, home care and hospice facility)

They also felt that appreciation was not always directed to the right people. The appreciation from society was often focused on the care provided in hospitals, compared to other healthcare settings such as nursing homes, and this was sometimes perceived as unfair, as a hospital nurse said about nursing home staff.

"So I did find that tricky in the first wave, all that applause, and we got an awful lot of gifts in that period, whereas I was thinking: hold on, if you work in a nursing home then people are dying who you know really well. Look, I don’t know the patients at all well, so in that sense you’re a bit further removed from it all." (R8: nurse, hospital)

This discrepancy in appreciation highlighted a broader issue of how certain healthcare settings, such as nursing homes and home care, were sometimes overlooked despite the challenges in these settings.

##### Applause and kind words are not enough

Nurses stated that applause and kind words were not enough. They indicated that they wanted to have appreciation from society in the form of financial compensation, as well as higher priority in the vaccination policy.

"I think it’s all very well, that applause, fantastic, but let’s be honest: it doesn’t pay the bills." (R16:nurse, ICU)

"Of course, because we’re physically close to the people, and we work with vulnerable people. So that [being late in getting vaccinated] feels a bit as if you’re being left in the lurch. Kind of: you’re doing really important work, and how good it is you’re all there, but on the other hand… right, I think we got our turn slightly before ordinary young Dutch people but not much before […] and that is of course quite strange." (R8: nurse, hospital)

This highlighted that while emotional gestures were appreciated, they needed to be complemented by practical forms of appreciation, such as financial and institutional backing, to fully meet the needs of healthcare workers during the prolonged crisis.

The government did spend money to show appreciation for nurses specifically a 1000 euro bonus. However, nurses noted that this form of appreciation, while financial, was not perceived as equitable or sufficient. This highlighted a disconnect between the public displays of appreciation and the real needs of nurses.

"You work yourself to the bone for two years and what do you get? Some kind of care bonus of a thousand euros that I think virtually the whole hospital got, even if they’d never seen a COVID patient." (R17:nurse, ICU).

The dissatisfaction with how appreciation was expressed also extended to the recognition nurses received from their employers. They felt valued when working conditions were good, appreciation was expressed verbally, and when their employer protected them from for instance overworking. Additionally, small gestures, organizing gatherings with colleagues, and acknowledging that it was a difficult time for them were also important for their sense of appreciation.

"And also having your team manager give you the space […] to look after yourself properly. Because the healthcare culture is one of […] doing a lot of stuff at home too, reading through files before coming into work. She said you shouldn’t be doing that, as it’s part of your job. So you don’t need to be doing that stuff at home already; you can just do all that when you get to work. Perhaps the clients then need to wait a bit longer, but so be it. But that’s also really nice as I think it shows appreciation." (R6: nurse, nursing home)

Some nurses described the desire of receiving small and thoughtful acts of appreciation. A hospital nurse expressed how she would have liked her employer to show appreciation:

"Yes, including the manager. I think: hey, you should be pleased with the people you still have, so why not just say that, just show them appreciation, express that. For example, you could buy a couple of cans of soft drinks with your own money and put them in the fridge. You know, tell us ‘I filled the fridge with some drinks, so after your shift you can stay on a bit for an evaluation together.’ Think up something. You know, make sure people feel they are appreciated." (R16:nurse, ICU)

In addition, it was also important for nurses to be involved in decision-making. Nurses sometimes felt that decisions were imposed on them by higher authorities without their own input, both at the employer and societal levels. When nurses were excluded from these processes, it reinforced the sense that their contributions were undervalued.

"Perhaps what I found most painful about this crisis was that an awful lot of decisions are taken for you at a higher level, I mean at the Executive Board level. They decide what specifically is going to change for you in the workplace. […] We really don’t have much of a say in that. […] That means we’re simply not involved at all in certain decisions, hospital-wide, and not at all in the Netherlands more broadly either. There was a lot of talk about the IC nursing staff, but nobody really ever talked to us." (R17:nurse, ICU)

#### We are not in this together

When nurses were asked if they felt appreciated, they often mentioned a deeper issue of not feeling understood by society, their own surroundings and sometimes even their employers. This misunderstanding reflected a disconnect between those experiencing the pandemic as healthcare providers and those observing healthcare from a distance. They felt only understood by people who were going through the same thing. They noticed that this lack of understanding manifested in societal behavior, such as people not following the general COVID-19 rules in place to slow the spread of the virus and that could lead to anger and frustration.

"I was tempted to give them all a smack in the chops, you know, all that ‘put a facemask on, keep your distance.’ […] That could make me so angry. […] That they don’t realise what those infections mean. And they don’t know what you’ve been through, they don’t know what you’ve seen. You get what I mean? That’s the thing. If you could only give them a glimpse… right, that they could see some of all that, then maybe they would have understood. But I don’t think you can ever explain it properly, no… " (R3:nurse aid, nursing home).

This disconnection was more than just about people breaking the general COVID-19 rules. In the workplace, they also noticed that people did not always follow the rules and sometimes engaged in arguments or even became aggressive. This was experienced as distressing by the nurses. Respondents noted that even if they did not personally experience anger and aggression in the workplace, they were affected by seeing or hearing about it happening to others.

"What you also heard then in the hospitals is that people were being confronted with aggression and so… So it’s not so much that I personally had to deal with that, but more how people from the outside treated you. […] For example, that people in the hospitals were treated badly by visitors and so on — that did affect me a bit. We didn’t experience that, but it makes me think gosh, that really is awful." (R12:nurse, home care and hospice facility)

Some nurses not only faced misunderstanding but also disbelief from people who did not believe that COVID-19 was real, that the consequences were severe, and that healthcare facilities were genuinely busy. This sometimes led to discussions, especially in the workplace. This disbelief from patients and the public further diminished the sense of appreciation for the emotional and professional efforts of nurses, making it difficult for them to continue providing care with the same level of compassion.

"I notice that people have less… well not less affection in caring for someone, but they do have a feeling where you’re caring for someone in a bed like that and you think: right, this person doesn’t believe in what we’re doing, so what exactly am I doing here?" (R7:nurse, nursing home)

When nurses discussed whether they felt appreciated, the conversation often quickly turned to people not following the basic government rules. Nurses indicated that they experienced the direct consequences of this, as it made their workload heavier, and it felt like not everyone in society was working towards the same goal as they were. The additional workload caused by society’s disregard for rules compounded the stress nurses already faced, and the lack of collective responsibility diminished their sense of appreciation for the hard work they were putting in. They mentioned that they were also tired of following the rules and would prefer to go back to their normal routines, but they recognized the necessity. The fact that society did not experience this and did not follow the rules made them feel like they were the ones suffering the negative consequences. A nurse from a nursing home even described it as a conflict:

"And yes, well, almost a conflict with the outside world, where everyone kept on asking for the rules to be relaxed and didn’t stick to the precautions and I don’t know what, whereas here in the nursing homes the best we could do was struggle to keep it at bay and stop our people getting sick. So yes, we’re all talking about various divisions in society back then due to Covid, but I reckon that was a pretty major division, between the outside world and the world inside the nursing homes." (R7:nurse, nursing home)

#### Short-lived appreciation that failed to lead to structural changes

Where nurses were almost overwhelmed with appreciation during the first wave (especially from society), the contrast afterwards was even greater. After the first wave, appreciation declined and sometimes even turned into anger and aggression. The sudden change in public sentiment, from applause to aggression, caused many nurses to feel a loss of appreciation, leaving them disillusioned with the temporary nature of the support.One nurse mentioned that the drastic change in public reactions compared to the first wave was one of the reasons she experienced burnout. Nurses also noticed a difference in their immediate surroundings:

"I found that a big difference too with the second wave. […] There was no understanding among the families any more, so you often got into discussions with people about the visiting rules. Everyone became bad-tempered and no one wanted to keep to the rules anymore; I thought that was a really big difference with the first time." (R16:nurse, ICU)

"Of course, if you look at it, last year people were standing there clapping and you were a hero — that kind of thing. And people used to ask you much more how you were doing. Say, if you happened to… neighbours, friends… well, now no one ever asks how you’re doing." (R2:nurse, ICU)

Nurses often found it difficult that during the first COVID wave there were many praiseful words from society and there seemed to be a lot of appreciation and attention to the well-being of healthcare providers, but nothing concrete changed in their situation to actually support them. For instance, that people just went on with their lives and that there were no actions to retain and attract healthcare staff.

"At the same time, I also think OK, they’re applauding us but then people go off and lead their lives as they want, kind of. I wonder how much we’ve all learned from this. […] And I do get a bit cynical about it sometimes. Then I think, you’ve had this pandemic […] and we all basically said how important it is that the healthcare should be good and you need the staff for this, but that doesn’t really lead to any action." (R8: nurse, hospital)

The absence of long-term changes made it clear that the appreciation shown at the start of the pandemic was shallow and didn’t last. Simultaneously with the COVID-19 pandemic, negotiations for a new collective labor agreement in academic hospitals were ongoing. The difficult progress and the disappointing outcome of these negotiations impacted nurses who worked in academic hospitals.

"The only thing that really hurts, and that I find really annoying, is that you all spend two years working your socks off and an awful lot of nurses appeared in current affairs programmes and there seemed to be so much respect, but when the collective labour agreement negotiations start, it all turns out very disappointing." (R1:nurse, hospital)These negotiations were a clear reminder of how little meaningful support had actually changed, despite all the public talk of gratitude and respect for healthcare workers.

## Discussion

This study shows that between September 2020 and September 2021, about 1 in 3 healthcare providers had a score on the Well-Being Index that indicated they were at greater risk for burnout, severe fatigue, poor overall quality of life, and moderate or greater intent to leave their current positions within the next 24 months. In nursing homes and hospitals, the well-being of healthcare providers was generally worse. Nearly half of healthcare providers felt misunderstood during the first 18 months of the pandemic, which was significantly associated with lower well-being. Furthermore, the feeling of being appreciated was highest in the first wave of the pandemic, but significantly dropped in the second wave, with only about half of the healthcare providers feeling appreciated. This feeling improved slightly after the second wave. When healthcare providers felt appreciated, it was significantly associated with better well-being. The interviews also revealed that nurses did not always feel understood and appreciated by society in general, their employers, patients and their families, as well as their own friends and family. This led to feelings of sadness, anger, and frustration. Three major themes emerged: ‘recognizing real needs, ‘we are not in this together’ and ‘short-lived appreciation that failed to lead to structural changes’.

### Recognizing real needs

This study confirms other research that describes appreciation and understanding as important for the mental well-being of healthcare providers [22, 23, 25, 26]. This study shows that appreciation and understanding operate on different levels, including society, the overarching setting in which they work (such as hospitals), their employers, the people they care for, and their own family and friends. When we asked about appreciation during the interviews, nurses often spoke about feeling misunderstood, suggesting a likely connection between the two concepts. It is also possible that these concepts are sometimes used interchangeably when discussing appreciation in the interviews. Interestingly, what nurses perceived as societal appreciation often did not (only) involve applause, kind words on social media, or their hero status. In fact, these gestures sometimes made them uncomfortable because they were simply doing their regular work, which was now suddenly seen as heroic. It is even suggested that this hero status can have a negative effect, as healthcare workers may find it insincere and see it as a way to prevent them from complaining about their poor working conditions, something heroes are not expected to do [41, 42]. All of the public appreciation paled in comparison to the feeling that they were not working together with society towards the same goal. They wanted practical support from society and politics to keep the workload low, specifically by following the rules. This is also described in a study on COVID-19-related moral injury among healthcare providers, which noted feelings of abandonment when demonstrations against regulations and vaccinations were held [43]. It is important to recognize the impact of appreciation on the well-being of healthcare workers, not only at a societal level but also in their interactions with relatives, patients, patients' relatives, and employers. Every group—whether personal or professional—has a responsibility to acknowledge the value of healthcare workers and to contribute to their well-being by showing appreciation. Addressing these issues at a structural level is vital not only for the present but also for future pandemic preparedness and the retention of healthcare workers, especially in contexts heavily impacted by the pandemic, such as end-of-life care. National policies aimed at addressing the sense of being misunderstood and unappreciated could improve the overall well-being of healthcare providers. This applies to situations such as a pandemic, but it also seems important for preventing healthcare providers from leaving due to mental health issues.

Also, the way healthcare providers felt appreciated by their employers varied. Although appreciation as a form of support is often mentioned in studies [27–31], there is little guidance on how to effectively show appreciation [29]. This study shows that while they certainly valued appreciation in terms of working conditions and verbal acknowledgment, there were also less obvious ways in which they felt appreciated. For example, when their employers protected them from overworking and recognized that the situation they were in was difficult, rather than only approaching it positively. To recognize that the situation is difficult, employers must also understand its specifics. Therefore, it is important that they show interest and observe the situation firsthand. This aspect is not often mentioned in studies but aligns with the findings of the current study that healthcare providers often feel misunderstood by those who do not experience it. Aside from these aspects that employers can implement, they may also have a role in helping healthcare workers navigate public opinion about their work or role.

### From applause to disappointment

It is interesting how the feeling of appreciation declined so much during the second COVID wave, which is evident both in the quantitative and qualitative data. The work that healthcare providers had always done suddenly became visible (for example, literally on TV) and crucial for society. Healthcare providers felt more recognized and hoped that this would finally be a turning point, leading to greater appreciation, more efforts to retain them, and making the profession more attractive for future healthcare providers. However, the shift from early applause to disappointment highlights a gap between initial public appreciation and long-term structural appreciation. This mismatch has implications for how future pandemics should be managed, particularly in terms of maintaining public engagement and ensuring sustained support for healthcare workers. There was disappointment when this did not happen, and when it shifted to more misunderstanding and aggression. Although some healthcare providers mentioned a kind of ‘us versus them’ feeling, where they spoke of a struggle with the outside world and their workplace, there was no explicit mention of feelings of stigmatization or exclusion, which other studies have highlighted [44–46]. Even though not all healthcare providers directly faced aggression, the increasing aggression toward their colleagues affected them. This is also described in a Latin American study, where nearly half of the healthcare providers who experienced some form of aggression (verbal to physical) reported psychosomatic symptoms after the event, and 1 in 3 considered leaving their profession [47]. Furthermore, the quantitative data shows a significant drop in the sense of appreciation during the second wave. This was confirmed in the interviews. Although the interviews did not indicate that appreciation increased again afterward, the quantitative data shows that healthcare providers did feel more appreciated after the second wave. It seems the significant contrast between the first and second waves might partly explain the initial decline between the first and second wave.

### Strengths and limitations

A strength of this study is its comprehensive overview of end-of-life care during the pandemic, as it encompasses multiple healthcare settings and professions. The longitudinal design, with data collected up to 18 months after the pandemic's onset, further enhances its thoroughness. Additionally, the mixed methods design allowed us to not only describe the impact of appreciation on well-being but also to use the qualitative data to clarify what respondents actually meant by appreciation. Due to COVID-19 measures, all interviews were conducted via (video) call. This could have affected the depth of the interviews by hampering building rapport with respondents. Despite this, we found respondents eager to share their experiences, and we believe the interviews maintained depth comparable to our usual face-to-face interactions. In the later stages of the study, no new topics emerged compared to earlier interviews. However, the complexity and rapid evolution of the COVID-19 situation across different healthcare settings mean we cannot be certain we reached saturation. This study may have underrepresented respondents in severe emotional distress, as they might have been less likely to complete the (follow-up) questionnaires. Consequently, those who were doing relatively well might be overrepresented, potentially leading to an underestimation of distress and therefor the need for support among health care providers. Additionally, there could have been recall bias in the initial questionnaire, which asked about an earlier time period. However, given the exceptional nature of the pandemic's onset, respondents might recall their feelings during that time quite accurately. Furthermore, we only included nurses in our interview study. We chose this approach to narrow our focus and because the initial survey indicated that nurses' well-being was most at risk. It would be interesting to investigate whether these interview results also apply to other healthcare providers.

## Conclusion

This study shows that there was a lack of understanding and appreciation for health care providers during the COVID-19 pandemic, which is associated with lower experiences of well-being among healthcare providers. The appreciation that was expressed often did not align with what healthcare providers needed or expected for their hard work. At a time when the well-being of healthcare providers is still under pressure and efforts must be made to create a sustainable situation for them, it is important to not forget the themes of appreciation and understanding. To retain healthcare providers, it is important that the attention and appreciation for them does not diminish.

## Supplementary Information

Supplementary Material 1.

Supplementary Material 2.

Supplementary Material 3.

Supplementary Material 4.

## Data Availability

The datasets used and/or analysed during the current study are available from the corresponding author on reasonable request.
