# Supplementary material for: From applause to disappointment – appreciation among healthcare providers that provided end-of-life care during the COVID-19 pandemic and its impact on well-being – a longitudinal mixed methods study (the CO-LIVE study)
Source: BMC Health Serv Res. 2024 Dec 18;24:1613. doi: 10.1186/s12913-024-11999-6 (PMC11653579; doi:10.1186/s12913-024-11999-6)
Supplement: Supplementary file 1 — Supplementary Material 1. [file 12913_2024_11999_MOESM1_ESM.docx]

| **Appendix 1: Original (translated) and recoded questions in the questionnaire** | | |
| --- | --- | --- |
| **Variable** | **Original question** | **Recoded** |
| **Gender** | **What is your gender?**   - Male - Female - Other - I would rather not say |  |
| **Age** | **What is your age?** | **Categorized**  *⩽35 years*  *36-45 years*  *46-60 years*  *>60 years* |
| **Profession** | **In what role were you caring for patients?**  Nursing assistant  Nurse  Physician assistant  General practitioner  Elderly care physician  Other medical specialty, namely:  ___________________________________  Other care professional, namely:  __________________________________  Volunteer | **Categorized**   - Nurse   *Nurse aides, nurse, physician assistant*   - Physician  *General practitioner, elderly care physician, physician with a different specialism* - Other  *Other healthcare professional, volunteer* |
| **Setting** | **In which setting did you provide care?**  *(more than one answer possible)*  At home / community  In a hospital  At an ICU  At a ward for Corona patients (no ICU)  At another ward  In a care home / nursing home  At a ward for Corona patients  At another ward  In a hospice facility (not specifically for Corona patients)  Other (please specify): _______________________________ | **Categorized**   - Home   *At home/community*   - Hospital   *In a hospital (ICU/at ward for COVID patients, at another ward)*   - In a care home / nursing home   *(At a ward for COVID patients, at another ward)*   - Hospice facility   *For COVID patients, not for COVID patients*   - Other   *Other*   - More than one |
| **Feeling appreciated** | Please rate to what extent you agree with the follow statements when you think about how you felt during [*earlier defined period]*?  I felt appreciated (as a healthcare worker).  Strongly disagree  Disagree  Neutral  Agree   Strongly agree  Don’t know | **Dichotomized**   - Neutral/disagree   *Neutral, strongly disagree, disagree*   - Agree   *Agree, strongly agree* |
| **Feeling not understood** | Please rate to what extent you agree with the follow statements when you think about how you felt during [*earlier defined period]*?  I have the feeling that people that did not work in healthcare did not understand me  Strongly disagree  Disagree  Neutral  Agree   Strongly agree  Don’t know | **Dichotomized**   - Neutral/disagree   *Neutral, strongly disagree, disagree*   - Agree   *Agree, strongly agree* |
| **Well-Being Index** | **In the last month…** | **Questions 1-7:**  - Answer ‘yes’: +1 point  **Question 8:**  - Option 1 & 2: +1 point  - Option 3-5: 0 points  - Option 6 & 7: -1 point  **Question 9:**  - Disagree & Strongly Disagree: +1 point  - Neutral: 0 points  - Agree & Strongly Agree: -1 point |
| **1** | Have you felt burned out from your work?  Yes  No |  |
| **2** | Have you worried that your work is hardening you emotionally?  Yes  No |  |
| **3** | Have you often been bothered by feeling down, depressed  Yes  No |  |
| **4** | Have you fallen asleep while sitting inactive in a public space?  Yes  No |  |
| **5** | Have you felt that all the things you had to do were piling up so high that you could not overcome them?  Yes  No |  |
| **6** | Have you been bothered by emotional problems (such as feeling anxious, depressed, or irritable)?  Yes  No |  |
| **7** | Has your physical health interfered with your ability to do your daily work at home and/or away from home?  Yes  No |  |
| **8** | Please rate to what extent you agree with the following statement:  The work I do is meaningful to me  (on a scale from 1 to 7)  1. Very strongly agree   2.  3.  4.  5.  6.  7. Very strongly sagree |  |
| **9** | Please rate to what extent you agree with the following statement:  My work schedule leaves/left me enough time for my personal/family time.  Mijn werkschema laat genoeg tijd over voor mijn privé/familieleven  Strongly agree   Agree  Neutral  Disagree  Strongly disagree |  |
