# Supplementary material for: From applause to disappointment – appreciation among healthcare providers that provided end-of-life care during the COVID-19 pandemic and its impact on well-being – a longitudinal mixed methods study (the CO-LIVE study)
Source: BMC Health Serv Res. 2024 Dec 18;24:1613. doi: 10.1186/s12913-024-11999-6 (PMC11653579; doi:10.1186/s12913-024-11999-6)
Supplement: Supplementary file 2 — Supplementary Material 2. [file 12913_2024_11999_MOESM2_ESM.docx]

**Appendix 2. Interview Topic List**

**Can you tell us about how your work has been since the outbreak of COVID-19?**

- How have you experienced this period?
- Specifically regarding end-of-life care
- Comparison with the first wave (T1) or the last time we spoke

**Can you give an example of a situation where you found end-of-life care challenging/touching/memorable during this period?**

- What made it that way?
- Did the situation present a dilemma?
  - Did this situation cause doubt about what the right thing to do was?
- How did you handle this situation?
- How do you handle dilemmas in your work?
- What helps you in these situations?

**Based on the 'complaints' in the survey (emotionally challenging, need for support)**

- Check if the situation from the survey is still accurate (e.g., in the survey you mentioned xx, yy, zz. Can you elaborate?)
  - Can you give an example?
  - Cause/causes?
  - To what extent has (the possible change in) your work affected your well-being?
  - How does it manifest?
  - How do you cope with it?
  - Why is it better or worse now?

**Coping**

- What kept/keeps you going?
- Why do some people cope better than others? What contributes to this?
- Who or what gives you support?

**Support**

- What do you need regarding to (emotional support)?
- Do you receive support (if you need it)?
  - What did that support look like and how did you find it?
- Positive or negative experiences?
- Comparison over time?
- How would you like the support to look?
  - What would you advise others regarding support?

**The following themes were explored in the survey. Based on the answers, respondents can be asked to explain further:**

- Misunderstanding by others
  - How do you notice this? (example)
  - How does that affect you?
- Appreciation
  - How do you notice this? (example)
  - From whom?
  - How does that affect you?

- Solidarity
