# Supplementary material for: From applause to disappointment – appreciation among healthcare providers that provided end-of-life care during the COVID-19 pandemic and its impact on well-being – a longitudinal mixed methods study (the CO-LIVE study)
Source: BMC Health Serv Res. 2024 Dec 18;24:1613. doi: 10.1186/s12913-024-11999-6 (PMC11653579; doi:10.1186/s12913-024-11999-6)
Supplement: Supplementary file 3 — Supplementary Material 3. [file 12913_2024_11999_MOESM3_ESM.docx]

| **Characteristics of interview respondents** | | | | | | | |
| --- | --- | --- | --- | --- | --- | --- | --- |
| **Rsp** | **Gender** | **Age** | **Profession** | **Setting** | **Interviews**  **T1 T2 T3** | | |
| **1** | Female | ⩽35 years | Nurse | Hospital | X |  |  |
| **2** | Female | 46-60 years | Nurse | ICU | X | X | X |
| **3** | Female | 36-45 years | Nurse aid | Nursing home | X | X | X |
| **4** | Female | 46-60 years | Nurse aid | Nursing home | X | X | X |
| **5** | Female | ⩽35 years | Nurse aid | Nursing home | X | X | X |
| **6** | Female | 46-60 years | Nurse | Nursing home | X | X |  |
| **7** | Female | 46-60 years | Nurse | Nursing home | X | X | X |
| **8** | Female | ⩽35 years | Nurse | Hospital | X | X | X |
| **9** | Female | 46-60 years | Nurse | Home care & nursing home | X | X | X |
| **10** | Female | 46-60 years | Nurse | Hospice facility | X | X | X |
| **11** | Female | >60 years | Nurse | Home care & hospice facility | X | X | X |
| **12** | Female | 46-60 years | Nurse | Home care & hospice facility | X | X | X |
| **13** | Female | ⩽35 years | Nurse aid | Nursing home | X | X | X |
| **14** | Female | 46-60 years | Nurse | Hospital | X |  |  |
| **15** | Female | ⩽35 years | Nurse | ICU |  | X | X |
| **16** | Female | 46-60 years | Nurse | ICU |  | X | X |
| **17** | Female | ⩽35 years | Nurse | ICU |  | X | X |

Appendix 3. Characteristics of interview respondents
