# Supplementary material for: From applause to disappointment – appreciation among healthcare providers that provided end-of-life care during the COVID-19 pandemic and its impact on well-being – a longitudinal mixed methods study (the CO-LIVE study)
Source: BMC Health Serv Res. 2024 Dec 18;24:1613. doi: 10.1186/s12913-024-11999-6 (PMC11653579; doi:10.1186/s12913-024-11999-6)
Supplement: Supplementary file 4 — Supplementary Material 4. [file 12913_2024_11999_MOESM4_ESM.docx]

| **Themes** | **Sub-codes** | **Codes** |
| --- | --- | --- |
| Recognizing real needs | Apprecation in the first wave | More recognition for work and well-being |
|  |  | Felt uncomfortable with apprecation |
|  |  | Apprecation was not directed to the right people |
|  | Applause and kind words are not enough | Vaccination policy |
|  |  | Financial compensation |
|  |  | Apprecation from employers |
|  |  | Being involved in decision-making |
| We are not in this together |  | Feeling not understood because people did not follow COVID rules in society |
|  |  | Discussion and aggression about COVID rules |
|  |  | Disbelievers |
|  |  | Conflict healthcare and society when following the rules |
| Short lived appreciation did not lead to structural changes |  | Changes in public reactions after the first wave |
|  |  | No actions to retain and attract healthcare staff |
|  |  | Disappointing collective labor agreement negotiations. |

**Appendix 4. Code scheme**
